# Demonstrating mood repair with a situation‐based measure of self‐compassion and self‐criticism

**DOI:** 10.1111/papt.12056

**Published:** 2015-02-05

**Authors:** Caroline J. Falconer, John A. King, Chris R. Brewin

**Affiliations:** ^1^ Department of Clinical, Educational & Health PsychologyUniversity College LondonUK

**Keywords:** self‐compassion, self‐criticism, feedback, mood repair

## Abstract

**Objectives:**

The clinical significance of self‐criticism and self‐compassion has prompted the development of questionnaires assessing these constructs. However, there is a lack of measures assessing their interaction within specific contexts and potential involvement in mood repair processes.

**Design:**

To rectify this, we developed the Self‐Compassion and Self‐Criticism Scales (SCCS), based on responses to specific scenarios, and examined its psychometric properties in an online survey and an experimental situation.

**Method:**

In study 1, standard psychometric procedures were used to investigate the reliability and validity of the SCCS. In study 2, an experimental challenge involving a difficult language task was used to test its sensitivity to change.

**Results:**

In study 1, exploratory factor analysis (*n* = 413) showed a clear two‐factor structure of the SCCS denoting two orthogonal scales, with high internal validity (α ≥ .87). Correlations between the SCCS and existing measures also demonstrated appropriate convergent validity. Study 2 (*n* = 90) provided preliminary evidence that the SCCS can detect changes in self‐appraisals. Participants receiving no performance feedback from the challenge task showed reduced state self‐criticism and increased state self‐compassion, demonstrating mood repair.

**Conclusions:**

The SCCS has promise as a situational measure of self‐compassion and self‐criticism.

**Practitioner points:**

- In the context of specific problem situations, clients' levels of self‐criticism and self‐compassion may well be orthogonal and can be assessed with the SCCS.
- In setting treatment goals and assessing treatment outcome, it may be helpful to target both self‐compassion and self‐criticism separately.

## Background

Adding to a long‐standing interest in self‐criticism, there has been an increased focus over the last decade in measuring self‐compassion, and several reliable and valid questionnaires of both constructs now exist (Blatt, D'Afflitti, & Quinlan, 1979; Brewin & Shapiro, 1984; Gilbert, Clarke, Hempel, Miles, & Irons, 2004; Neff, 2003b). However, there is a lack of research addressing how processes of self‐compassion and self‐criticism interact at specific moments in time or under specific circumstances. Coinciding with this gap in the research is an absence of measures that assess both self‐compassion and self‐criticism in parallel. This study sought to develop and validate a new measure incorporating both constructs, with the ultimate aim of furthering understanding of the dynamic relationships between them. This new measure is the first of its kind and its potential research and clinical applications in the form of experimental state assessment and online therapy monitoring will significantly contribute to the progression of the field.

Excessive self‐criticism is one of the most significant psychological processes thought to influence the susceptibility to and the maintenance and relapse of many psychopathologies (Brewin & Firth‐Cozens, 1997; Hewitt & Flett, 2002; Ingram, 2003; Koerner & Linehan, 1996; Pagura, Cox, Sareen, & Enns, 2006; Southwick, Yehuda, & Giller, 1995). Maladaptive self‐criticism can be defined as a persistent tendency for negative self‐evaluation that instils feelings of shame and low self‐worth. The aetiology of chronic self‐criticism is thought to arise early in life through a lack of or deficient affiliative relationships (Andrews, 1995; Andrews, Brewin, Rose, & Kirk, 2000; Brewin, Firth‐Cozens, Furnham, & McManus, 1992; Koestner, Zuroff, & Powers, 1991; Sachs‐Ericsson, Verona, Joiner, & Preacher, 2006).

Equally important for psychopathology is a deficit in the ability to self‐soothe and reassure, which is also thought to arise from inadequate nurturing during childhood (Gilbert, 2010b) and can amplify negative self‐appraisals (Gilbert, 2010a,b). Compassionate self‐soothing and reassurance has been regarded as our natural regulator of shame and self‐criticism (Gilbert, 2010a; Leary, Tate, Adams, Allen, & Hancock, 2007; Neff, 2003b; Neff & Vonk, 2009). Indeed, self‐compassion is a good predictor of positive affect (PA) and happiness, is associated with more enduring feelings of self‐worth (Neff & Vonk, 2009), and predicts coping in the face of failure and stress (Neely, Schallert, Mohammed, Roberts, & Chen, 2009; Neff, Hsieh, & Dejitterat, 2005). Developing and enhancing self‐compassion through training has significantly reduced self‐criticism, shame, and depression in chronically depressed patients and has even improved psychological well‐being in healthy individuals (Leary *et al*., 2007; Neff & Germer, 2012).

One of the very few studies to investigate self‐criticism and self‐compassion simultaneously was that of Longe *et al*. (2010), who investigated neural activity during responses to threatening scenarios. However, although fMRI data suggested critical and compassionate responses are, at least partially, independent, subjective responses to each of the scenarios were not assessed. Future studies would benefit from a standardized state questionnaire including both constructs, to investigate short‐term dynamic interactions between them and their associations with changes in situation and mood.

In our view, self‐compassion and self‐criticism are complex cognitive, emotional, motivational, and behavioural responses to the self that have specific temporal relationships and may be difficult for individuals to assess in the abstract. Merely asking the statement ‘to what extent do you currently feel self‐compassionate’ does not take into account this complex process. For this reason, we developed the Self‐Compassion and Self‐Criticism Scales (SCCS) around imagined scenarios, inspired by Longe *et al*. (2010). Self‐compassion was operationalized as self‐kindness or self‐reassurance, consistent with Gilbert (2010a,b). Scenarios and their self‐referential responses were established through discussions with colleagues in the clinical psychology field. Negative self‐referential items were selected based on common descriptions of negative self‐relating experienced by clinical colleagues in discourse with depressed patients. Positive items were generated from common adjectives of compassionate, positive self‐relating promoted during therapy (Gilbert, 2010a). In study 1, the SCCS was piloted and the factor structure and internal validity of responses examined within and across the scenarios. In study 2, we tested the sensitivity of the SCCS to experimental manipulations and its relation to changes in different types of affect.

## STUDY 1

## Method

### Participants

In total, 413 participants took part in this study (254 females). The majority of participants were aged 18–24 years (62.6%) followed by 19.9% aged 25–34 years, 10.2% aged 45–54 years, 5.1% aged 35–44 years, and 2.2% aged 55–64 years. Participants were recruited through an online survey that was advertised throughout the university and on social networking sites. Participants were given a URL link to the survey and provided informed consent to participate. Demographic information was recorded including age and sex. In addition to the SCCS, participants completed several other questionnaires assessing trait self‐compassion and self‐criticism, which are outlined below. Debriefing information was provided at the end of the study. Questionnaire presentation and data collection were accomplished using the Qualtrics online survey platform and the order of questionnaire presentation was randomized across participants. Experimental procedures were approved by the University's School of Psychology Ethics Committee.

### Measures

#### Self‐Compassion and Self‐Criticism Scales

The initial version of the SCCS consisted of eight self‐threatening scenarios that can elicit varying degrees of self‐criticism or self‐compassion:

1. A third job rejection letter in a row arrives in the post.
2. You arrive after walking to a meeting to find that you are late and the doors are closed.
3. You arrive home to find that you have left your keys at work.
4. You receive a letter in the post that is an unpaid bill reminder.
5. You have just dropped and scratched your new Smart phone.
6. You have just received a failed test result.
7. You have just opened the washing machine door to find that your white wash has turned pink.
8. After searching your bag you realize that you have lost a £20 note.

On a 7‐point Likert Scale (1 = not at all; 7 = highly), participants rated the extent to which they would react to themselves in a Harsh, Contemptuous, Hostile, Cold, Critical, Soothing, Reassuring, Compassionate, and Warm manner in relation to each imagined scenario.

#### Forms of Self‐Criticizing/Attacking and Self‐Reassuring Scale

The Forms of Self‐Criticizing/Attacking and Self‐Reassuring Scale (Gilbert *et al*., 2004) is a measure of trait self‐criticism and self‐reassurance. On a 5‐point Likert Scale, participants rate the extent to which various statements are true of them (1 = not at all like me; 5 = extremely like me). The questionnaire comprises three scales: Inadequate self (IS: e.g., ‘I am easily disappointed’), hated self (HS: e.g., ‘I stop caring about myself’), and reassured self (RS: e.g., ‘I find it easy to forgive myself’). Authors reported Cronbach's alphas of .90 for the IS and .86 for the HS and RS scales.

#### Trait Self‐Criticism Scale

This scale was developed by Brewin *et al*. (1992) and is a combination of items from the Depressive Experiences Questionnaire (Blatt *et al*., 1979) and the scale of Responsibility for Negative Outcomes (Brewin & Shapiro, 1984), both of which have acceptable reliability and validity. This 9‐item scale is concerned with trait self‐criticism and the perception of blameworthiness regarding life outcomes. Participants rate on a 7‐point Likert Scale the extent to which they agree or disagree (1 = Strongly Disagree; 7 = Strongly Agree) with statements of trait self‐criticism (‘I often find that I don't live up to my own standards or ideals’) and self‐blame (‘My misfortunes have resulted mainly from the mistakes I've made’). Authors reported a Cronbach's α of .83.

#### Trait Self‐Compassion Scale

This scale (Neff, 2003b) measures six aspects of trait self‐compassion and includes 26 items rated on a 5‐point Likert Scale of frequency (1 = almost never; 5 = almost always). The author reported Cronbach's alphas of .75–.81 for subscales and .92 for the whole scale. In view of recent evidence that the subscales are independent and do not measure a single overarching compassion construct (Williams, Dalgleish, Karl, & Kuyken, 2014), this study utilized the self‐kindness subscale as the closest conceptually to the SCCS (e.g., ‘I'm kind to myself when I'm experiencing suffering’).

## Results

### Exploratory factor analysis on the SCCS

We first conducted an exploratory factor analysis (EFA) of the response items for each of the eight scenarios to examine the factor structure and to establish item retention. A principal components method with direct oblimin rotation was used to allow any factors to correlate. Each scenario EFA showed a strong Kaiser–Meyer–Olkin measure of sampling adequacy (>.76) and a significant Bartlett's test of sphericity (*p *<* *.001). A two‐factor structure was revealed for each scenario, as indicated by Eigenvalues >1. The first factor of each scenario included all of the self‐critical items with factor loadings ranging from .67 to .89. The percentage of explained variance from this factor ranged between 33.3% and 40.3% across scenarios. The second factor for each scenario was comprised of the self‐compassionate items with factor loadings ranging from .72 to .92. The percentage of explained variance from this factor ranged between 23.3% and 33.3% across scenarios. The combined explained variance of both factors ranged between 63% and 70% across scenarios. With the exception of scenario 1 (*r *=* *−.23), the two factors did not significantly correlate with one another (*p *>* *.05).

Two additional EFAs were conducted to establish the nature of any inherent factor structure among the scenarios. The scenarios were analysed with separate summed whole scores from the self‐compassion and self‐criticism factors established in the first EFA. The EFA showed a strong Kaiser–Meyer–Olkin measure of sampling adequacy (.94) and a significant Bartlett's test of sphericity (*p *<* *.001) for the self‐compassion factor across scenarios. The self‐criticism factor also had a strong Kaiser–Meyer–Olkin measure of sampling adequacy (.91) and a significant Bartlett's test of sphericity (*p *<* *.001). The EFA revealed a one‐factor structure from the scenarios for both self‐compassion and self‐criticism responses. The factor loadings ranged from .68 to .85 for the self‐compassion responses accounting for 66% of the explained variance. Factor loadings for the self‐criticism responses ranged from .75 to .81 and accounted for 62% of the explained variance.

### Scale reduction

To keep testing time to a minimum, we reduced the overall number of response items to three self‐compassion and three self‐criticism items and reduced the scenarios from 8 to 5. The State Self‐Compassion Scale is composed of four response items, of which ‘Warm’ consistently had the lowest factor loading across all of the scenarios and was therefore removed from the scale. The Self‐Criticism Scale is composed of five response items of which ‘Cold’ consistently had the lowest factor loading across all scenarios and was also removed. The next lowest loadings were for ‘Critical’ and ‘Hostile’, which were very similar, and we decided to remove ‘Hostile’. As the State Self‐Compassion Scale includes the item ‘Compassionate’, the inclusion of the item ‘Critical’ maintained the comparability of the scales. An additional EFA and internal validity testing was conducted on the reduced items in each scenario. This reaffirmed a two‐factor structure of state self‐criticism and self‐compassion and showed that the percentage of explained variance increased across the scenarios.

Reducing the number of scenarios was initially determined by the factor loading of each scenario in contributing to the whole scores of the State Self‐Compassion and the State Self‐Criticism Scales. Scenario 1 loaded the least for both the scales and was removed as a result. Scenario 2 was the second scenario to be removed as this was present in the lowest three scenario loadings in both scales. Scenarios 6 and 7 were also present in the lowest three factor loadings for the SCCS, respectively. We removed scenario 6 as this had the greatest skew and least variance for the State Self‐Compassion Scale. EFAs were conducted to establish whether the removal of these three scenarios influenced the factor structure among the remaining scenarios. The scenarios were analysed again with separate summed whole scores from the SCCS. The EFA showed a strong Kaiser–Meyer–Olkin measure of sampling adequacy (.94) and a significant Bartlett's test of sphericity (*p *<* *.001) for the State Self‐Compassion Scale across scenarios. The State Self‐Criticism Scale also had a strong Kaiser–Meyer–Olkin measure of sampling adequacy (.89) and a significant Bartlett's test of sphericity (*p *<* *.001). The analysis confirmed a one factor structure for each of the scales, with increased percentage of explained variance (Table 1). Cronbach's alpha was good for both the State Self‐Criticism Scale (α = .87) and for the State Self‐Compassion Scale (α = .91). A complete version of the SCCS and instructions are available as a file in Data S1.

**Table 1:** Factor loadings and descriptive statistics for Self‐Compassion and Self‐Criticism Scales across scenarios

Scenario	Self‐compassion	Self‐criticism
	Loadings	Loadings
5. You have just dropped and scratched your new smart phone	.86	.82
8. After searching your bag you realize that you have lost a £20 note	.86	.82
4. You receive a letter in the post that is an unpaid bill reminder	.86	.79
7. You have just opened the washing machine door to find that your white wash has turned pink	.85	.81
3. You arrive home to find that you have left your keys at work	.83	.81
Cronbach's α	.91	.87
Mean	35.1	53.2
*SD*	16.8	18.6

### Demographic associations

There was no significant difference between male scores (*M *=* *33.9, *SD* = 15.5) and female scores (*M *=* *35.9, *SD* = 17.6) for the State Self‐Compassion Scale, *t*(409) = −1.12, *p *=* *.26. There was also no significant difference found between male scores (*M *=* *53.5, *SD* = 18.9) and female scores (*M *=* *53.1, *SD* = 18.6) for the State Self‐Criticism Scale, *t*(409) = 0.696, *p *=* *.86. Linear contrasts showed that age was not associated with either State Self‐Criticism or Self‐Compassion Scales (*p *>* *.05).

### Convergent validity

Table 2 presents the correlations between the self‐compassion and self‐criticism scores of the SCCS and the five pre‐existing scales of trait self‐criticism and self‐compassion. As expected, the state self‐criticism scores are positively correlated with HS, IS, and trait self‐criticism scores, and negatively correlated with RS scores.

**Table 2:** SCCS self‐compassion and self‐criticism correlations with existing measures

	SCCS self‐compassion	SCCS self‐criticism	Trait self‐criticism^a^	HS	IS	RS	Self‐kindness^a^
SCCS self‐compassion	1	.026	−.070	.011	−.055	.132**	.206**
SCCS self‐criticism		1	.256**	.176**	.299**	−.120*	−.074
Trait self‐criticism^a^			1	.522**	.743**	−.433**	−.258**
HS				1	.543**	−.518**	−.323**
IS					1	−.449**	−.291**
RS						1	.603**
Self‐kindness^a^							1
Mean	35.1	53.2	36.4	8.8	27.8	27.5	2.9
*SD*	16.8	18.6	11.3	4.1	7.7	5.9	.76

Note

HS, Hated Self; IS, Inadequate Self; RS, Reassured Self; SCCS, Self‐Compassion and Self‐Criticism Scales.

^a^ *N *=* *291.

*p <* *.05; **p <* *.01.

State self‐compassion scores did not significantly correlate with HS, IS, or trait self‐criticism scores but, as predicted, there were positive correlations with the RS score (*r *=* *.13, *p *=* *.007) and with self‐kindness (*r *=* *.21, *p *<* *.001). Self‐compassion did not correlate significantly with the overall trait self‐compassion scores (*p *=* *.48).

## Discussion

This study demonstrates a clear two‐factor structure of the SCCS denoting two separate scales of state self‐criticism and self‐compassion, both of which have excellent internal validity. Furthermore, the study provides evidence for convergent validity with positive correlations between the new State Self‐Criticism Scale and existing measures of trait self‐criticism, and negative correlations with the existing measure of self‐reassurance. There were also positive correlations between the State Self‐Compassion Scale and existing measures of self‐reassurance and self‐kindness. Importantly, state self‐criticism and self‐compassion did not correlate with one another when measured within the same scenarios. This contrasts with the moderate negative correlations between these constructs on existing measures requiring agreement with more general written statements. Given that state levels of self‐criticism and self‐compassion from the SCCS do not correlate with one another, it is not surprising that there is a lack of or weak correlation between the scales and their opposing trait measurements. These data suggest that the two processes of state self‐criticism and self‐compassion may be independent, and not opposite ends of a bipolar construct, within the context of specific situations. Thus, the format of the SCCS may provide additional flexibility in assessing possible dynamic interactions between self‐criticism and self‐compassion.

Study 2 was designed to assess whether the SCCS can adequately measure change in levels of state self‐compassion and self‐criticism in response to negative affect (NA) prompted by different types of task feedback. Affective responses to feedback are known to depend not just on degree of success and failure but on idiosyncratic causal attributions made for the outcome (Weiner, 1985), which are more difficult in the absence of feedback. As lack of feedback creates uncertainty and NA in its own right (Epstein, 1972), no prediction was made about whether NA would be greater in response to low performance feedback or a no feedback control condition. We hypothesized that state self‐compassion and self‐criticism would change differentially as a function of NA generated by feedback on task performance. Thus, study 2 was also designed to address the relationship between state affect and SCCS scores, and to confirm that changes in state self‐criticism and self‐compassion could not be reduced to changes in NA and PA, respectively.

## STUDY 2

## Method

### Participants

Ninety participants took part in this study (56 females), none of whom took part in study 1. Ninety per cent of the participants were aged 18–24 years and 10% were aged 25–34 years. Experimental procedures were approved by the University's School of Psychology Ethics Committee.

### Materials and measures

Participants were required to complete a difficult antonym task. This antonym task was developed from the language section of the American Graduate Record Examination, similar to Breines and Chen (2012). The task involves the presentation of a target word and a list of five possible antonyms. The participant has to choose the correct antonym (e.g., ‘glut’) that corresponds to the target word (e.g., ‘dearth’). There were 25 randomized trials in total. In addition to the antonym task, participants completed the SCCS and two additional mood questionnaires.

#### International Positive and Negative Affect Schedule, Short Form (I‐PANAS‐SF)

Positive and negative affect were measured with the 10‐item I‐PANAS‐SF (Thompson, 2007), a cross‐culturally reliable and briefer version of the original PANAS (Watson, Clark, & Tellegen, 1988). Participants rated how strongly they were currently experiencing a particular emotion on a 5‐point Likert Scale (1 = not at all; 5 = very much so) (e.g., PA: Active, inspired; NA: Ashamed, hostile). Cronbach's alphas for the PA and NA scales are .78 and .76, respectively (Thompson, 2007).

#### Two Forms of Positive Affect Scale

The Two Forms of Positive Affect Scale (TFPAS) measures the extent to which participants experience 18 different positive emotions (Gilbert *et al*., 2008) forming three types of PA: Active Affect (e.g., energetic), Relaxed Affect (e.g., calm) and Safe Affect (e.g., content). This scale allows for a better approximation of affect systems associated more specifically with self‐compassion (Gilbert, 2010a; Gilbert *et al*., 2008). Participants rate on a 5‐point Likert Scale (1 = not at all; 5 = very much so) how strongly they are experiencing these emotions at the current moment in time. Authors reported a Cronbach's α of .83 for Active and Relaxed Affect, and .73 for Safe Affect Scales.

### Procedure

Participants were initially told that the purpose of the experiment was to investigate the influence of mood on language processing. This was to ensure that any attempts at guessing the hypothesis would not bias participant responses. After providing consent, participants completed the I‐PANAS‐SF, SCCS, and the TFPAS. Participants then completed the antonym task, after which they were randomly provided either with feedback in the form of a high or low percentage of correct responses (84% and 28%, respectively) or no feedback. No information about normative levels of performance on the test was provided so that participants did not know whether their indicated level of performance was good or bad. They then completed the three questionnaires for a second time. Finally, they were asked to write down the purpose of the experiment and the extent to which they found the antonym task difficult (7‐point Likert Scale where 1 = ‘very difficult’; 7 = ‘very easy’).

### Data processing

The data were inspected for outliers 2.5 standard deviations above and below the mean but none were identified. Scores for the SCCS, I‐PANAS‐SF, and the TFPAS sub‐scales were all positively skewed and therefore log‐transformed to normalize their distribution. Data were analysed using 7 separate 2 × 3 mixed‐model analyses of variance (ANOVA) with Time (before, after) as the within‐subjects variable and Feedback (low, high, no feedback) as the between‐subjects variable. Sex was entered into the ANOVA as an additional between‐subjects variable, but no significant sex effects were found in any analysis (*p* > .05) unless indicated below. *Post‐hoc*, pairwise comparisons were Bonferroni corrected.

## Results

### Difficulty ratings and performance

A one‐way ANOVA showed that there was no statistical difference between the three feedback conditions on the perceived difficulty of the antonym task, *F*(2, 87) = 1.23, *p *=* *.29. The mean score was 2.25 (*SD* = 1.1) indicating a high degree of perceived difficulty across all three groups. There was also no statistical difference between the three feedback conditions in terms of percentage of correct responses, *F*(2, 87) = 1.6, *p* = .207. In addition, performance (mean percentage of correct response = 48.8%, *SD* = 13.4%) did not correlate with change scores of state self‐compassion and self‐criticism (*p *>* *.1) for all three groups, indicating that performance was unrelated to changes in self‐relating.

### SCCS self‐compassion

Significant effects were found for Time, *F*(1, 87) = 6.88, *p *=* *.010, $\eta_{p}^{2}$ = .073, indicating an overall increase, and Feedback, *F*(2, 87) = 3.72, *p *=* *.028, $\eta_{p}^{2}$ = .079, as well as their interaction, *F*(2, 87) = 5.62, *p *=* *.005, $\eta_{p}^{2}$ = .115. *Post‐hoc* pairwise comparisons revealed a significant increase in self‐compassion scores after no feedback (mean difference = 5.9, *p *=* *.001), which was not the case for low (mean difference = 0.77, *p *=* *.57) or high (mean difference = 1.2, *p *=* *.34) percentage score feedback. Table 3 provides a summary of study 2 results.

**Table 3:** SCCS means and (*SD*) at testing time points for feedback groups

	High percentage score feedback		Low percentage score feedback		No feedback	
	Time 1	Time 2	Time 1	Time 2	Time 1	Time 2
SCCS self‐criticism	58.9 (15.5)	57.7 (17.1)	57.6 (14.3)	58.5 (17.2)	53.5 (16.7)	48.2 (15.5)**
SCCS self‐compassion	33.8 (13.4)	35.0 (14.5)	30.8 (14.1)	30.0 (12.8)	37.5 (16.9)	43.5 (16.1)**
PANAS positive	15.0 (4.2)	14.0 (4.6)	14.2 (3.5)	12.7 (3.3)**	15.8 (3.3)	11.8 (3.1) **
PANAS negative	9.6 (4.3)	8.7 (3.6)	9.7 (4.1)	10.5 (4.1)	9.6 (4)	13.4 (3.5)**
Active affect	12.8 (5.1)	12.8 (5.6)	10.8 (4.6)	8.4 (5.2)**	12.2 (4.9)	12.6 (5)
Relaxed affect	14.6 (5.5)	14.7 (5.9)	13.4 (4.3)	12.0 (5.5)	13.3 (4.4)	8.0 (3.8)**
Safe affect	10.5 (3.8)	10.6 (4.1)	9.5 (3.3)	8.7 (3.6)	10.0 (3.0)	6.9 (2.8)**

Note

PANAS, International Positive and Negative Affect Schedule; SCCS, Self‐Compassion and Self‐Criticism Scales.

**Significant difference from time point 1, *p *<* *.01. There were no significant differences in Time 1 measurements across the three feedback groups, *p *>* *.21.

### SCCS self‐criticism

A significant main effect was found for Time, *F*(1, 87) = 4.49, *p *=* *.037, $\eta_{p}^{2}$ = .049, indicating an overall decrease in self‐criticism scores, but not for Feedback, *F*(2, 87) = 2.23, *p *=* *.113, $\eta_{p}^{2}$ = .049. There was a significant interaction between Time and Feedback, *F*(2, 87) = 4.29, *p *=* *.017, $\eta_{p}^{2}$ = .090. *Post‐hoc* pairwise comparisons revealed a significant reduction in self‐criticism scores after no feedback (mean difference = 5.4, *p *=* *.005), which was not the case for low (mean difference = 0.93, *p *=* *0.54) or high (mean difference = 1.2, *p *=* *.36) percentage score feedback (*p *>* *.05). When sex was entered into the ANOVA as a between‐subjects variable, there were no significant sex effects found (*p *>* *.05) with an exception of a three‐way interaction between sex, feedback condition and time point, *F*(2, 84) = 5.96, *p *=* *.004, $\eta_{p}^{2}$ = .124.

### I‐PANAS‐SF positive affect

A significant main effect was found for Time, *F*(1, 87) = 45, *p *<* *.001, $\eta_{p}^{2}$ = .341, indicating a decrease from time point 1 to 2, but there was no effect of Feedback, *F*(2, 87) = 0.35, *p *=* *.70, $\eta_{p}^{2}$ = .008. A significant interaction between Time and Feedback condition was found, *F*(2, 87) = 7.9, *p *=* *.001, $\eta_{p}^{2}$ = .155. *Post‐hoc* pairwise comparisons revealed a significant decrease in PA scores after no feedback (mean difference = 4.0, *p *<* *.001) and after low percentage score feedback (mean difference = 1.4, *p *=* *.012), but not after high percentage score feedback (mean difference = 0.93, *p *=* *.07).

### I‐PANAS‐SF negative affect

A significant main effect was found for Time, *F*(1, 87) = 10.31, *p *=* *.002, $\eta_{p}^{2}$ = .106, indicating an increase in NA scores at time point 2. There was a significant main effect of Feedback, *F*(2, 87) = 3.84, *p *=* *.025, $\eta_{p}^{2}$ = .081, that was qualified by a significant interaction between Time and Feedback, *F*(2, 87) = 11.4, *p *<* *.001, $\eta_{p}^{2}$ = .208. *Post‐hoc* pairwise comparisons revealed a significant increase in NA scores after no feedback (mean difference = 3.8, *p *=* *.001), which was not the case for low (mean difference = 0.87, *p *=* *.109) or high (mean difference = 0.93, *p *=* *.049) percentage score feedback. When sex was entered into the ANOVA, there was a significant main effect, *F*(1, 84) = 7.47, *p *=* *.008, $\eta_{p}^{2}$ = .082, revealing a higher score for men (*M *=* *11.2, *SD* = 4.1) than women (*M *=* *9.5, *SD* = 3.81).

### TFPAS active affect

No significant main effect was found for Time, *F*(1, 87) = 1.97, *p *=* *.17, $\eta_{p}^{2}$ = .02, indicating no overall decrease in active affect. There was a significant main effect of Feedback condition, *F*(2, 87) = 4.4, *p *=* *.016, $\eta_{p}^{2}$ = .09, which was qualified by a significant interaction, *F*(2, 87) = 3.28, *p *<* *.042, $\eta_{p}^{2}$ = .07. *Post‐hoc* pairwise comparisons revealed a significant decrease in active affect scores after low percentage score feedback (mean difference = 2.33, *p* = .005), which was not the case for high score percentage (mean difference = 0.03, *p* = .96) and no feedback (mean difference = 0.40, *p* = .62) conditions.

### TFPAS relaxed affect

A significant main effect was found for Time, *F*(1, 87) = 28.4, *p *<* *.001, $\eta_{p}^{2}$ = .25, indicating a decrease in relaxed affect scores, and for Feedback, *F*(2, 87) = 5.9, *p *=* *.004, $\eta_{p}^{2}$ = .12, qualified by a significant interaction, *F*(2, 87) = 15.4, *p *<* *.001, $\eta_{p}^{2}$ = .26. *Post‐hoc* pairwise comparisons revealed a significant decrease in relaxed affect scores after no feedback (mean difference = 5.33, *p *<* *.001), which was not the case for low (mean difference = 1.37, *p *=* *.06) and high (mean difference = 0.07, *p *=* *.92) percentage score feedback conditions.

### TFPAS safe affect

A significant main effect was found for Time, *F*(1, 87) = 21.5, *p *<* *.001, $\eta_{p}^{2}$ = .20, indicating an overall decrease in safe affect, and for Feedback, *F*(2, 87) = 3.39, *p *=* *.038, $\eta_{p}^{2}$ = .07. There was a significant interaction between Time and Feedback, *F*(2, 87) = 12.7, *p *<* *.001, $\eta_{p}^{2}$ = .23. *Post‐hoc* pairwise comparisons revealed a significant decrease in safe affect scores after no feedback (mean difference = 3.1, *p *<* *.001), which was not the case for low (mean difference = 0.83, *p *=* *.08) and high (mean difference = 0.17, *p *=* *.72) score percentage feedback.

## GENERAL DISCUSSION

Study 2 provides preliminary evidence that the SCCS is sensitive to change. The difficult antonym task elicited a reduction in PA in the low percentage feedback condition, consistent with the well‐established association between outcomes and emotions (Weiner, 1985). The absence of more marked emotional reactions was probably due to the lack of normative information on which to base causal attributions for their performance. Such attributions are the major determinant of achievement‐related emotion (Weiner, 1985). More widespread reductions in PA and increases in NA were observed in the no feedback condition, consistent with the predictions of Epstein (1972) concerning the effect of uncertainty on anxiety. These were accompanied by compensatory increases in state self‐compassion and decreases in self‐criticism.

This pattern of results, with increased compassion accompanying negative mood changes, is reminiscent of other examples of mood repair processes in the literature (Josephson, 1996; Power & Brewin, 1990; Sanchez, Vazquez, Gomez, & Joormann, 2014). For example, Power and Brewin (1990) presented participants with the names of hypothetical life events and required them to indicate whether or not a subsequent trait adjective was self‐descriptive. In the face of an esteem‐threatening life event, participants were slower to endorse negative adjectives and overall endorsed fewer such adjectives as self‐descriptive. Josephson (1996) reported that participants low in previous levels of depression were more likely to respond to a sad mood induction by retrieving positive memories on a subsequent autobiographical memory task. Finally, Sanchez *et al*. (2014) found that participants' choice to fixate happy rather than sad faces after a negative mood induction predicted mood recovery at the end of the experiment.

The association between NA, increased state self‐compassion, and decreased state self‐criticism, although consistent with the mood repair literature, is in contrast with results from questionnaire studies, which generally show that higher levels of PA are positively correlated with trait self‐compassion and negatively correlated with trait self‐criticism, whereas the opposite is true for NA (Gilbert & Irons, 2004; Gilbert *et al*., 2004; Neff, 2003b; Neff & Vonk, 2009). On the surface, these previous studies might lead one to expect increased PA in the presence of increased state self‐compassion and reduced state self‐criticism. Questionnaire‐based studies cannot, however, capture the dynamic aspects of self‐compassion and self‐criticism and the way in which they respond to situational determinants. This will necessarily limit the amount of validity that can be obtained from such measures. Our data underscore the value of using multiple methods of investigation including the provision of opportunities to repair negative moods.

There have been no systematic investigations into whether levels of trait self‐criticism and self‐compassion vary as a function of age in adulthood, but there is some evidence to suggest that this may be the case (Allen, Goldwasser, & Leary, 2011; Gilbert *et al*., 2004; Neff & Pommier, 2012). This should be taken into account when using the SCCS, because the majority of our validating samples were young adults.

New studies are emerging that investigate cultural differences in trait self‐criticism and self‐compassion (Ghorbani, Watson, Chen, & Norballa, 2011; Neff & McGehee, 2010; Wong & Mak, 2013; Yamaguchi & Kim, 2013). Self‐compassion has been conceptualized, partially, within the framework of Buddhist psychology (Neff, 2003a,b), which could suggest increased levels of self‐compassion in Far Eastern societies. Neff, Pisitsungkagarn, and Hsieh (2008) have shown cultural differences between East and West, with increased levels of self‐compassion in Thailand as compared to the United States of America. However, they also show variations in self‐compassion among Far Eastern countries, indicating potentially complex interactions between self‐compassion and culture. It is important to note that the present study did not assess cultural differences. However, the SCCS does permit future exploration of cultural differences at a situational level.

There is also evidence in the literature showing sex differences in trait self‐criticism and self‐compassion (Kupeli, Chilcot, Schmidt, Campbell, & Troop, 2013; Neff, 2003b; Neff & Pommier, 2012; Neff & Vonk, 2009). Our data did not reveal significant differences between males and females. However, there were 95 fewer men than women in our validating sample, which could have influenced these results. Future research should aim to replicate this finding and investigate the potential discrepancies between different questionnaire methodologies as a function of sex.

While the SCCS has not yet been validated within clinical populations, it could be used to investigate similarities and differences between clinical and non‐clinical populations in their tendency to respond self‐critically or self‐compassionately in specific situations. Indeed, Falconer *et al*. (2014) have further demonstrated the sensitivity of the SCCS as a state measure. A brief compassion intervention with highly self‐critical participants in virtual reality was shown to selectively reduce state self‐criticism and increase self‐compassion scores on the SCCS. The cultivation of self‐compassion through therapy is currently only assessed by changes in trait measures after several weeks of therapy (Gilbert & Irons, 2004; Gilbert & Procter, 2006; Mayhew & Gilbert, 2008; Neff & Germer, 2012). The SCCS could also be useful to investigate the progress of individuals across therapy sessions. Additionally, the SCCS might be useful in predicting the occurrence of state self‐compassion and self‐criticism when patients are faced with specific challenging situations. Furthermore, we are currently investigating the potential integration of personalized scenarios within the SCCS, which may offer a more patient centred assessment of treatment.

## Supporting information


**Data S1.** Self‐Criticism and Self‐Compassion Scale (SCCS).
