# Supplementary material for: Demonstrating mood repair with a situation‐based measure of self‐compassion and self‐criticism
Source: Psychol Psychother. 2015 Feb 5;88(4):351–65. doi: 10.1111/papt.12056 (PMC4949512; doi:10.1111/papt.12056)
Supplement: Supplementary file 1 — Data S1. Self‐Criticism and Self‐Compassion Scale (SCCS). [file PAPT-88-351-s001.docx]

Self-Criticism and Self-Compassion Scale (SCCS)

Below are several statements describing various situations. Accompanying each statement is a list of possible reactions that you may have in response to **yourself** during these situations. We would like you to rate on the scales the extent to which you would react to **yourself** in a specific manner in response to each situation, *as if the situation were happening to you at this moment in time*. Try to imagine each situation occurring as vividly as possible.

1. “You arrive home to find that you have left your keys at work”

Not at All Highly

Reassuring 1 2 3 4 5 6 7

Soothing 1 2 3 4 5 6 7

Contemptuous 1 2 3 4 5 6 7

Compassionate 1 2 3 4 5 6 7

Critical 1 2 3 4 5 6 7

Harsh 1 2 3 4 5 6 7

1. “You receive a letter in the post that is an unpaid bill reminder”

Not at All Highly

Reassuring 1 2 3 4 5 6 7

Soothing 1 2 3 4 5 6 7

Contemptuous 1 2 3 4 5 6 7

Compassionate 1 2 3 4 5 6 7

Critical 1 2 3 4 5 6 7

Harsh 1 2 3 4 5 6 7

1. “You have just dropped and scratched your new Smartphone”

Not at All Highly

Reassuring 1 2 3 4 5 6 7

Soothing 1 2 3 4 5 6 7

Contemptuous 1 2 3 4 5 6 7

Compassionate 1 2 3 4 5 6 7

Critical 1 2 3 4 5 6 7

Harsh 1 2 3 4 5 6 7

1. “You have just opened the washing machine door to find that your white wash has turned pink”

Not at All Highly

Reassuring 1 2 3 4 5 6 7

Soothing 1 2 3 4 5 6 7

Contemptuous 1 2 3 4 5 6 7

Compassionate 1 2 3 4 5 6 7

Critical 1 2 3 4 5 6 7

Harsh 1 2 3 4 5 6 7

1. “After searching your bag you realise that you have lost a £20 note”

Not at All Highly

Reassuring 1 2 3 4 5 6 7

Soothing 1 2 3 4 5 6 7

Contemptuous 1 2 3 4 5 6 7

Compassionate 1 2 3 4 5 6 7

Critical 1 2 3 4 5 6 7

Harsh 1 2 3 4 5 6 7
